# Using a partially randomized patient preference study design to evaluate the therapeutic effect of acupuncture and cupping therapy for fibromyalgia: study protocol for a partially randomized controlled trial

**DOI:** 10.1186/1745-6215-15-280

**Published:** 2014-07-10

**Authors:** Hui-Juan Cao, Jian-Ping Liu, Hui Hu, Nissi S Wang

**Affiliations:** 1 Beijing University of Chinese Medicine, Bei San Huan Dong Lu 11, Chaoyang District, Beijing 100029, China; 2 Pamir Communications, Daly City, CA, USA

**Keywords:** Partially randomized patient preference, Fibromyalgia, Acupuncture, Cupping therapy, Feasibility study, Traditional Chinese medicine

## Abstract

**Background:**

Conducting randomized controlled trials on traditional Chinese non-drug therapies has been limited by factors such as patient preference to specific treatment modality. The aim of this study is to investigate the feasibility of applying a partially randomized patient preference (PRPP) trial model in evaluating the efficacy of two types of traditional Chinese medicine therapies, acupuncture and cupping, for fibromyalgia while accounting for patients’ preference of either therapeutic modality.

**Methods:**

This protocol was approved by the Institutional Ethics Committee of affiliated Dongfang Hospital, Beijing University of Chinese Medicine (approval number: 2013052104-2). One hundred participants with fibromyalgia will be included in this study. Diagnosis of fibromyalgia will be based on the American College of Rheumatology criteria. Before treatment, participants will be interviewed for their preference toward acupuncture or cupping therapy. Fifty participants with no preference will be randomly assigned to one of the two groups and another 50 participants with strong preference to either acupuncture or cupping will receive what they choose. For acupuncture and cupping therapy, the main acupoints used will be tender points (*Ashi).* Treatment will be three times a week for 5 consecutive weeks with a follow-up period of 12 weeks. Outcome measures will be qualitative (patient expectation and satisfaction) and quantitative (pain intensity, quality of life, depression assessment).

**Trial registration number:**

NCT01869712 (in clinicaltrials.gov, on 22nd May 2013).

## Background

In the last two decades, randomized controlled trials (RCTs) have been used to evaluate the efficacy of traditional Chinese medicine (TCM) therapies. In most cases, these trials have investigated herbal drugs [1]. However, limitations exist when conducting RCTs for non-herbal therapies such as acupuncture, cupping therapy, and moxibustion. Therapeutic effects can be influenced by factors other than the efficacy of the interventions themselves;such actors may include patient preference, practitioner preference, and patient-practitioner relationship, among others [2]. Based on the design principle of RCTs, participants are precluded from making their own choices during allocation to the intervention or control arms to avoid performance bias due to strong preference of participants who are not blinded during the trial. Unfortunately, there is no ideal placebo control for most types of non-drug therapies, which means successful blinding is almost impossible for these types of trials.

RCTs for non-drug therapies also have limitations associated with patient recruitment and adherence due to their preference of treatment [3]. Patients who visit TCM hospitals seeking non-drug treatment are likely to prefer such therapy. This not only increases the difficulty of recruiting for RCTs (especially for the control group) but also may lead to a high dropout rate during the trial.

A partially randomized, patient preference (PRPP) trial has been recommended for use in trials with potential performance bias [4-6]. This model was first applied for evaluating the therapeutic effect of surgery as compared with drugs, in which blinding methods could not be used due to the obvious inconformity between intervention and control treatment. Currently, this type of trial is widely used in studies that assess the effectiveness of a non-drug treatment (such as surgery) [7-11]. Given the limitations of a classical RCT, the PRPP model may be more suitable for evaluating non-drug TCM therapies. To date, there is no report on such trials in China to assess non-drug TCM therapies. Thus, our study is aimed at exploring the feasibility of applying the PRPP model to appraise the therapeutic effect of two kinds of non-drug therapies, acupuncture and cupping, while accounting for patient preference.

For this PRPP trial, we have chosen fibromyalgia as the target disease for three reasons. First, as a disorder with nonspecific symptoms of chronic widespread musculoskeletal pain and stiffness [12,13], fibromyalgia appears to affect increasing numbers of people and has a detrimental impact on their quality of life [14]. Second, acupuncture has been widely used for treating this condition [15]. Third, for pain conditions, the primary outcome is usually defined as pain relief as measured by a pain intensity instrument, such as a visual analog scale, which is based on patients’ subjective reporting and may be affected by belief, expectation, or preference of treatment modality.

Many kinds of TCM non-drug modalities are used to treat fibromyalgia. Acupuncture appears to have the most benefit in improving the main symptoms of fibromyalgia compared with herbal remedies [16-20]; thus, it is often recommended as an alternative therapy for this disorder [21]. Cupping therapy is an ancient Chinese healing modality. After systematically searching and analyzing clinical studies published in the last 50 years that used cupping therapy as the main intervention, we found that 70 of 550 studies evaluated cupping therapy for pain conditions [22]. From these studies, it appears that cupping therapy is effective for pain reduction in general, but few studies looked at cupping therapy for fibromyalgia. Our report [23] on medicinal cupping for fibromyalgia found that, after cupping therapy, the visual analogue scale (VAS) scores of 30 participants were reduced by 52.3% from baseline and the number of tender points was 30.9% fewer than in baseline. In China, acupuncture and cupping therapy are commonly used in TCM hospitals for pain management, but there have been no trials comparing these two treatment modalities for fibromyalgia.

### Objectives

The objectives of this trial are to assess the feasibility of applying the PRPP trial model in evaluating the therapeutic effect of acupuncture versus cupping, and to observe and compare the efficacies of these two therapies for fibromyalgia.

## Methods

This protocol was registered at the U.S. National Institute of Health ClinicalTrials.gov (identifier: NCT01869712; http://clinicaltrials.gov/ct2/show/NCT01869712?term=NCT01869712&rank=1). The protocol was last updated on 15 October 2013.

### Study design

**Figure 1:**
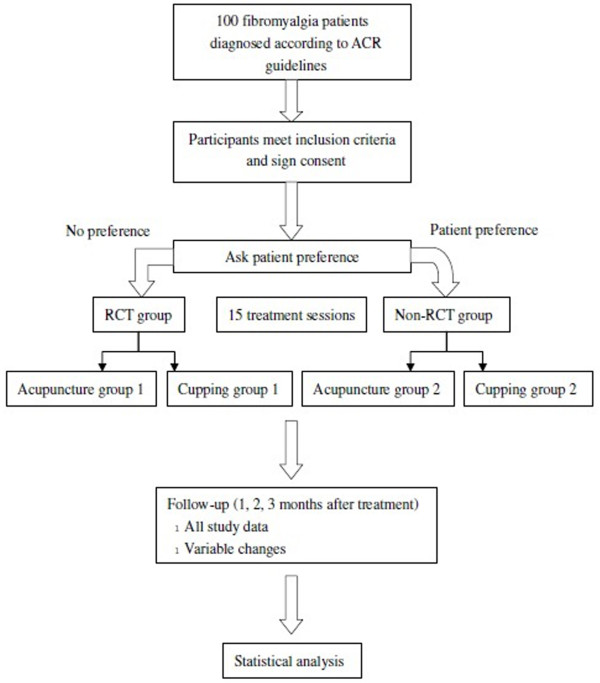
Study flow chart.

This will be a partially randomized patient preference (PRPP) trial with four parallel groups: randomized cupping therapy, non-randomized cupping therapy, randomized acupuncture, and non-randomized acupuncture. Outcome assessors and statisticians will be blinded (Figure 1).

In this study design, participants without preference to a treatment modality will be randomly divided into either the cupping or acupuncture group. Participants with strong preferences will be assigned to their modality of choice. The PRPP design not only enables comparisons between participants with and without a preference and an exploration of participant characteristics associated with preference, but also enables evaluation of total therapeutic effect of the two modalities, while accounting for participant preference.

#### Setting and recruitment

The study will take place at the outpatient department of acupuncture and moxibustion, Affiliated Dongfang Hospital, Beijing University of Chinese Medicine, China. Outpatients will be recruited. Recruitment advertising will be via posters and flyers distributed in the hospital and nearby communities, as well as through the Internet. To increase adherence and maintain retention during the trial, five complimentary sessions of acupuncture or cupping therapy will be provided for all included participants after the trial.

#### Estimate of sample size

The first aim of this trial will be to investigate the feasibility of applying the PRPP model to evaluate the therapeutic effect of TCM non-drug therapy. The primary outcomes will be participant adherence and satisfaction. As a feasibility study, there is no previous study that provides relative data for us to use to calculate sample size.

The second aim of this trial will be to observe the therapeutic effect of the two types of TCM non-drug therapies of acupuncture and cupping for fibromyalgia with an equivalent test hypothesis. For this objective, the outcome will be pain relief, which will be measured by the VAS. We have used G*Power (version 3.1.2) to calculate the sample size.The analysis of variance (ANOVA) repeated measures technique was used as the statistical method during sample size calculation and will also be used for data analysis. To estimate the effect of cupping, we used data from our previous study [22], in which cupping produced a decrease in pain of 12.78 (SD = 5.88) points on the 100-mm VAS. We also used data from a trial on acupuncture for fibromyalgia [24] to estimate changes in pain that might occur in the acupuncture group. This corresponded to an estimated effect size of .2875. Using a two-tailed test with an alpha rate of .05, this meant that 38 participants are required per group to achieve 80% statistical power. Thus, we will aim for a total of 76 participants in two groups. Allowing for a dropout of 15%, we will recruit a total of 100 participants.

#### Generation of the allocation sequence of randomization

Fifty patients who have no preference for either of the two therapies (acupuncture or cupping) will be randomly allocated to either the cupping or the acupuncture group. We will use SAS 8.0 software to create the sequence of randomization [25], total number of cases is 50, number of groups is 2, distribution ratio of the two groups is 1:1.

This sequence of randomization will be contained in opaque sealed envelopes and kept by researcher A, who will be contacted by each practitioner to provide the allocation.

#### Blinding methods

As the comparisons in this trial are different, outcome assessors and statistical analysts will be blinded. Outcome assessors will not be involved in participant allocation or treatment and will not be permitted to ask participants about information on allocation or treatment.

### Participants

#### Diagnostic criteria of fibromyalgia

Fibromyalgia will be diagnosed based on criteria formulated by the American College of Rheumatology (ACR) [26]. Key symptoms will be chronic widespread musculoskeletal pain, which will be assessed using scores of a widespread pain index (WPI) and a symptom severity (SS) scale [26].

#### Inclusion criteria

1. Participants with fibromyalgia diagnosed based on ACR criteria;

2. Participants whose scores for pain intensity are more than 30 mm (measured by VAS);

3. Participants 20 through 60 years of age;

4. Participants who fully understand the research process and are willing to provide informed consent.

#### Exclusion criteria

1. Persons with general musculoskeletal pain due to non-fibromyalgia illness;

2. Persons with mental disorders or other serious organic diseases, such as organ failure;

3. Persons taking oral pain medication or undergoing other interventions for pain relief during the trial;

4. Pregnant or lactating women;

5. Persons currently participating in another clinical trial.

#### Approach for cases of dropout/lost to follow-up/need to screen

1. Dropout cases will be defined as enrolled participants who miss a treatment session or refuse treatment as scheduled. We will consider at least 80% of the planned recruitment (40 cases in each group) to provide sufficient data for analysis.

2. Lost to follow-up will be defined as participants who complete all treatments, but cannot be contacted for follow-up.

3. Need to screen will be defined as enrolled participants who have serious adverse events during the trial. Researchers will be expected to halt treatment immediately and note the reason for the adverse event.

### Interventions

#### Cupping group (randomized and non-randomized)

Cups: Glass cups of three sizes will be used: large, 7 cm (edge diameter) × 9 cm (bore diameter) × 7.5 cm (height); medium, 6 cm × 8 cm × 6 cm; small, 6 cm × 7 cm × 5.3 cm. Jar size will be chosen based on treatment site.

Acupoints: *Ashi* (tender) points will be selected as the primary acupoints for flash cupping and retained cupping. Bladder channel (BL) and/or Du channel (DU) will be selected for moving cupping.

Cupping method: Retained cupping will be applied as the primary cupping method, supplemented with flash cupping and/or moving cupping. The procedure will be as follows. Suction (vacuum) will be created in the cups using flaming heat. Cups will then be applied immediately to acupoints on the BL and/or DU channels. Cups will be moved back and forth along the channel(s) several times. After cups are removed, flaming heat will be used to again create vacuum in the cups, which will then be applied to target treatment sites and retained for 10 minutes during each session. Treatment will be once daily, every other day, three sessions per week for five weeks.

#### Acupuncture group (randomized and non-randomized)

Needles: Stainless steel disposable needles (Wujiang Cloud Dragon Medical Instrument Company, Ltd., Jiangsu, China) of three lengths will be used, depending on the depth of the acupoint site: 0.25 mm × 25 mm, 0.25 mm × 40 mm, and 0.30 mm × 75 mm.

Acupoints: *Ashi*acupoints will be selected.

Acupuncture procedure: The skin at the acupoints will be disinfected. Needles will be inserted and manipulated for 15 seconds. Needle manipulation will be accomplished by using a twirling and up and down movement. Needles will be retained for 30 minutes each session. Treatment will be once daily, every other day, three sessions per week for five weeks.

#### Other allowable interventions

Patients will be permitted to take analgesics for pain relief during the follow-up period. Researchers will be expected to note the names, times, and doses of such medications.

### Outcome measures

#### Primary outcome

1. Patient adherence: Participant dropout,participants lost to follow-up, and reasons will be recorded (Table 1).

**Table 1:** Schedule for treatment and outcome measurement

**Item**	**Screening**	**Treatment**															**Follow-up**		
**Session**	**1 (baseline)**	**2**	**3**	**4**	**5**	**6**	**7**	**8**	**9**	**10**	**11**	**12**	**13**	**14**	**15**	**16**	**17 (+1 m)**	**18 (+2 m)**	**19 (+3 m)**
Eligibility screening	√																		
Informed consent	√																		
Patient preference screening	√																		
Allocation	√																		
Patient expectation toward treatment	√																		
Treatment		√	√	√	√	√	√	√	√	√	√	√	√	√	√	√			
Widespread pain index	√					√					√					√	√	√	√
Symptom severity	√					√					√					√	√	√	√
Visual analogue score	√					√					√					√	√	√	√
Fibromyalgia intensive questionnaire	√															√	√	√	√
Hamilton depression scale	√															√	√	√	√
SF-36 Health survey	√															√	√	√	√
Patient satisfaction of treatment																√			
Number of dropouts or lost to follow-up																√			√
Practitioner attitude toward research model																√			√
Recording of adverse events	√	√	√	√	√	√	√	√	√	√	√	√	√	√	√	√	√	√	√

2. Patient satisfaction for the treatment: Patient satisfaction will be assessed at week 5 (Table 1). Satisfaction will be measured using a seven-score scale, with 1 for “very satisfied” and 7 for “very dissatisfied”.

3. Practitioner attitude for the research model: Individual or focus group interviews will be conducted after the trial (Table 1). All practitioners in the study (acupuncturists and cupping therapy practitioners) will be interviewed for their disposition toward conducting the PRPP trial model and their thoughts on this model compared with traditional randomized controlled trials.

#### Secondary outcomes

1. Adverse events: Adverse events will be observed during the five weeks of treatment and three months follow-up (Table 1). Researchers will be expected to note minor and serious adverse events that occur, including case ID number and symptoms.

2. Patient expectation for the treatment: Patient expectation will be assessed at week 0 (Table 1), measured by a four-score scale, with 1 for “symptoms totally disappeared” and 4 for “no change in symptoms”.

3. Visual analogue scale (VAS) [27] for pain intensity: The length of the VAS we will use is 100 mm, with “0” representing no pain and “100” representing unbearable pain that affects appetite and quality of sleep. Researchers will be expected to show the scale to patients and note the pain scores. VAS for pain will be measured once daily during week 0, at the end of every fifth treatment session (for a total of 15 assessments during the five-week treatment period), then during weeks 9, 13, and 17 of the follow-up period (Table 1).

#### Other outcomes

1. Widespread pain index (WPI) for pain intensity: The WPI is defined based on the ACR diagnostic criteria for fibromyalgia [26]. The WPI will be measured once daily during week 0, at the end of every fifth treatment session (for a total of 15 assessments during the five-week treatment period), then during weeks 9, 13, and 17 of the follow-up period (Table 1).

2. Symptom severity (SS) for pain intensity: SS is defined based on the ACR diagnostic criteria [26] for fibromyalgia. SS will be measured once daily during week 0, at the end of every fifth treatment session (for a total of 15 assessments during the five-week treatment period), then during weeks 9, 13, and 17 of the follow-up period (Table 1).

3. Hamilton Depression Scale (HAMD; Chinese translation) for depression [28]: The HAMD will be implemented three times at weeks 0, 5, and 17 (Table 1).

4. Quality of life measured by the SF-36 Health Survey (Chinese translation) [29]: The SF-36 will be implemented three times, at weeks 0, 5, and 17 (Table 1).

5. Revised Fibromyalgia Impact Questionnaire (FIQR, Chinese translation) for pain intensity [30]: The FIQR will be implemented three times, at weeks 0, 5, and 17 (Table 1).

### Data handling and statistical analysis

#### Data collection

A case report form (CRF) for each enrolled participant, listing demographic characteristics and baseline symptom spectrum, data outcome at each visit (Table 1), adverse events, and analgesic use, will be completed by outcome assessors at each visit.

#### Data management

EpiData Software (version 3.0.2, The EpiData Association, Odense, Denmark) will be employed for data management. Data from CRFs will be entered and stored by two data entry staff members. Data entry accuracy will be ensured using the double entry method.

#### Statistical analysis

To evaluate the feasibility of the PRPP model, we will use the chi-square, Cochran-Mantel-Haenszel (CMH)-chi-square, or Fisher’s exact test (as appropriate) to compare patient adherence and satisfaction toward treatment between groups and qualitative research methods to analyze patient and practitioner attitudes.

To assess the therapeutic effect of acupuncture and cupping therapy for fibromyalgia, we will use the chi-square, CMH-chi-square, or Fisher’s exact test (as appropriate) to compare baseline characteristics for categorical data. Analysis of variance (normal distribution) or non-parametric tests (skewed distribution) will compare baseline characteristics for continuous data. Analysis of primary outcomes will involve contrast analysis in which cupping therapy will be compared with acupuncture. This will indicate whether or not cupping and acupuncture differ in efficacy. This analysis will be conducted using repeat measurements in which outcomes will be compared at each follow-up, controlling for baseline scores. Intention-to-treat analysis[31] will be used in which missing data will be supplemented using the value that was assessed at the prior session (for example, if participant drops out at treatment session 6, then all outcome data would be noted as values at session 5). All calculations will be performed using SPSS for Windows (version 17.0, Chicago, Illinois), and *P*-values under .05 will be considered significant.

### Ethical issue

This protocol, including the informed consent document and case report form, has been approved by the Institutional Ethics Committee of Beijing University of Chinese Medicine Affiliated Dongfang Hospital (approval number: 2013052104-2). All participants will be required to sign the informed consent form. Participant names will appear as pinyin abbreviations on CRFs. Only the practitioner who will oversee enrollment and outcome assessors will be aware of participants’ personal information, which will not be shared with other researchers and practitioners.

## Discussions

The average age of onset of fibromyalgia is 45 years with morbidity increasing with age [32]. Our systematic review of randomized controlled trials on TCM treatment for fibromyalgia found that the average age of patients was 40 to 50 yrs [20]. Thus, for this trial we will include persons aged 20 to 60 years, in order to focus on this high-risk group.

Researchers of this study include professionals in epidemiology, evidence-based medicine, biostatistics, and clinicians, all of whom will ensure the quality of design, application, and evaluation of this clinical trial. All acupuncturists and cupping therapists will be screened for licensure in traditional Chinese medicine and clinical experience of at least one year.

## Trial status

This study has been in the active recruitment phase since July 2013.

## Abbreviations

BL: bladder channel; CRF: case report form; FIQR: Revised Fibromyalgia Impact Questionnaire; HAMD: Hamilton Depression Scale; PRPP: partially randomized patient preference; RCT: randomized controlled trial; SD: standard deviation; SS: symptom severity; TCM: traditional Chinese medicine; VAS: visual analogue scale; WPI: widespread pain index.

## Competing interests

The authors declare that they have no competing interests.

## Authors’ contributions

CHJ provided the conception and design, coordinated contributions from the co-authors, and provided draft and final approval of the manuscript. LJP participated in the design of the study and in critical revision and final approval of the manuscript. HH provided conception and design, manuscript writing, and final approval of the manuscript and responded to the clinical comments of the reviewers. NSW provided conception and design, and critical revision and final approval the manuscript. All authors read and approved the final manuscript.
